# Superiority of Epstein-Barr Virus DNA in the Plasma Over Whole Blood for Prognostication of Extranodal NK/T Cell Lymphoma

**DOI:** 10.3389/fonc.2020.594692

**Published:** 2020-11-30

**Authors:** Joo Young Ha, Hyungwoo Cho, Heungsup Sung, Ah Ra Jung, Yoon Sei Lee, Sang-Wook Lee, Jin-Sook Ryu, Eun Jin Chae, Kyung Won Kim, Jooryung Huh, Chan-Sik Park, Dong-Joon Kim, Seon-Ok Kim, Dok Hyun Yoon

**Affiliations:** ^1^ Department of Oncology, Asan Medical Center, University of Ulsan College of Medicine, Seoul, South Korea; ^2^ Division of Hematology/Medical Oncology, Department of Internal Medicine, Chung-Ang University College of Medicine, Seoul, South Korea; ^3^ Department of Laboratory Medicine, Asan Medical Center, University of Ulsan College of Medicine, Seoul, South Korea; ^4^ Department of Otolaryngology, Asan Medical Center, University of Ulsan College of Medicine, Seoul, South Korea; ^5^ Department of Radiation Oncology, Asan Medical Center, University of Ulsan College of Medicine, Seoul, South Korea; ^6^ Department of Nuclear Medicine, Asan Medical Center, University of Ulsan College of Medicine, Seoul, South Korea; ^7^ Department of Radiology, Asan Medical Center, University of Ulsan College of Medicine, Seoul, South Korea; ^8^ Department of Pathology, Asan Medical Center, University of Ulsan College of Medicine, Seoul, South Korea; ^9^ Department of Clinical Epidemiology and Biostatistics, Asan Medical Center, University of Ulsan College of Medicine, Seoul, South Korea

**Keywords:** non-Hodgkin lymphoma, extranodal natural killer T cell lymphoma, plasma EBV-DNA, whole-blood EBV-DNA, prognosis

## Abstract

**Background:**

Extranodal natural killer T cell lymphoma (ENKTL) is a rare subtype of non-Hodgkin lymphoma with invariable infection of lymphoma cells with Epstein-Barr virus (EBV), and the presence of EBV-DNA in the blood is a well-known prognosticator. However, there is no consensus on which blood compartment is more optimal for predicting survival outcomes.

**Methods:**

We analyzed 60 patients who were newly diagnosed with ENKTL from a prospectively collected database. EBV-DNA was measured in the whole-blood (WB) and plasma at the time of diagnosis and after treatment completion.

**Results:**

EBV-DNA was detected in pre-treatment WB and plasma in 37 (61.7%) and 23 (38.3%) patients, respectively. The presence of pre-treatment plasma EBV-DNA was significantly associated with advanced stage while presence of WB EBV-DNA did not. Positivity of pre-treatment plasma-EBV, but not WB EBV-DNA, was independently associated with poor PFS (HR, 4.22;95% CI, 1.79–9.97; *P*=0.001) and OS (HR, 8.38; 95% CI, 3.03–23.19; *P*<0.001) in the multivariate analysis. After treatment completion, positivity of plasma-EBV was independently associated with poor PFS (HR, 9.41; 95% CI, 2.27–39.02; *P*=0.002) and OS (HR, 32.38; 95% CI, 3.25–322.56; *P*=0.003), whereas no significant association was observed between WB-EBV status and survival outcomes.

**Conclusions:**

Our results suggest that EBV-DNA in the plasma has better prognostic values than WB in patients with ENKTL.

## Introduction

Extranodal natural killer T-cell lymphoma (ENKTL) is a rare and distinct subtype of non-Hodgkin lymphoma characterized by predominant involvement of the nasal cavity and nasopharynx, and invariable infection of lymphoma cells with Epstein-Barr virus (EBV) (1). The recent introduction of combined chemotherapy and radiotherapy for localized disease (2–4) and the development of L-asparaginase-based chemotherapy has significantly improved the survival outcomes of patients with ENKTL; however, the prognosis of these patients still remain poor (2, 5).

The presence of EBV-DNA in the peripheral blood has been widely used to estimate the tumor burden of ENKTL (6) as the level of EBV-DNA at diagnosis correlates well with survival outcomes and treatment response (1, 7–9). Prognostic index for NK/T-cell lymphoma (PINK) model is one of the most widely accepted prognostic models for patients with ENKTL treated with non-anthracycline-based therapy, and the addition of EBV-DNA data to PINK (PINK-E) is useful in identifying the high-risk patients with poor survival outcomes (8). In addition, the post-treatment presence of EBV-DNA has also been shown to predict the risk of treatment failure in patients with ENKTL (10, 11).

Circulating EBV-DNA in the blood is derived from necrotic or apoptotic tumor cells and can be measured in the whole blood (WB) or plasma (12–14). A recent study has reported that there are discrepancies in the detection rates of EBV-DNA according to the types of blood compartments, and suggested that plasma samples are better than WB for evaluating or tracking response to therapy for EBV-positive diseases (15). In addition, a growing body of evidence shows that plasma EBV-DNA is the optimal marker for predicting survival outcomes in EBV-associated lymphoid malignancies such as Hodgkin’s lymphoma (16). However, there is no consensus on which blood compartment is more useful for measuring EBV-DNA for predicting survival and treatment outcomes in patients with ENKTL. Accordingly, PINK-E incorporates EBV-DNA status regardless of sample type. We thus evaluated and compared the prognostic values of plasma EBV-DNA and WB EBV-DNA in patients with ENKTL.

## Methods

### Patients

Baseline characteristics data were retrieved from the prospectively collected database, in which 60 patients with newly diagnosed ENKTL were consecutively enrolled from September 2014 to September 2018 in Asan medical center, Seoul, South Korea. The diagnosis of ENKTL was based on pathological and immunohistochemical examinations in accordance with the World Health Organization (WHO) classification (17). Real-time quantitative PCR for EBV-DNA was performed using both WB and plasma samples prior to treatment in all patients, and post-treatment in a subgroup of patients. Treatment responses were assessed by post-treatment CT scans and PET-CT scans at least 4 weeks after completion of the planned treatment according to the response criteria for non-Hodgkin lymphoma (18). This study was approved by the Institutional Review Board of Asan Medical Center.

### Quantification of WB and Plasma EBV-DNA

Peripheral blood samples were collected in EDTA tubes, and QIAamp® DNA mini kit (QIAGEN) was used for DNA extraction. The extracted DNA was eluted with 50 μl of elution buffer and quantified. EBV DNA load was quantified using the artus^®^ EBV LightCycler Kit (QIAGEN, Hilden, Germany). In accordance with the manufacturer’s instructions, the limit of detection level was 2.66 log copies/ml.

### Statistical Analysis

Categorical variables were evaluated using a chi-squared test or Fisher’s exact test, as appropriate. Shapiro-Wilk normality test was used to test for assumption of normal distribution of continuous variables, and the Student’s t-test or the Wilcoxon rank-sum test was used for comparisons of continuous variables between two groups, as appropriate. In addition, Bonferroni correction as post-hoc test was used for comparisons among three or more groups. Pearson correlation analysis was used to investigate the correlation between clinical and laboratory continuous variables. Overall survival (OS) was defined as the time from the start date of treatment to the date of death from any cause. Progression-free survival (PFS) was defined as the time from the start date of treatment to the date of relapse, progression, or death whichever occurred first. Survival analysis was performed using the Kaplan–Meier method, and comparisons were calculated using the log-rank test. Multivariate Cox regression analysis was used to estimate the prognostic impact of different variables on PFS and OS. Two-sided p values < 0.05 were considered statistically significant. Statistical analyses were performed using SPSS 22.0 (SPSS Inc., Chicago, IL, USA) and R version 3.6.0 (R Foundation for Statistical Computing, Vienna, Austria). Results are reported according to Reporting Recommendations for Tumor Marker Prognostic Studies (REMARK) guidelines (**Table S1**) (19).

## Results

### Patients and Treatment

The demographic and clinical characteristics of the 60 patients according to the presence of pre-treatment WB and plasma EBV-DNA are shown in **Table 1**. The median age was 55 years (range, 19–80); 29 (48.3%), 6 (10.0%), and 25 (41.7%) patients were in stages I, II, and IV, respectively. Twenty-seven (45.0%) patients received concurrent chemoradiotherapy followed by systemic chemotherapy and 26 (43.3%) patients received systemic chemotherapy alone. All of the 53 patients received L-asparaginase-based systemic chemotherapy. Patients who were unfit for systemic chemotherapy underwent concurrent chemoradiotherapy without consolidation chemotherapy (n=6) or radiotherapy alone (n=1). After treatment completion, 52 patients were available for response evaluation: 33 (63.5%), 9 (17.3%), and 10 (19.2%) patients had achieved complete response (CR), partial response (PR), and progressive disease (PD), respectively.

**Table 1:** Clinicopathological characteristics of patients at diagnosis according to the status of whole blood and plasma Epstein-Barr virus (EBV)-DNA (n = 60).

Characteristics (*n*, %)	Totalpatients	Pre-treatmentWBEBV-DNA (-)	Pre-treatmentWBEBV-DNA (+)	*P-*value	Pre-treatmentplasmaEBV-DNA (-)	Pre-treatmentplasmaEBV-DNA (+)	*P-*value
**No. of patients**	60 (100)	23 (38.3)	37 (61.7)		37 (61.7)	23 (38.3)	
**Age, median (range)**	55 (19–80)	59.0 (30-80)	55.0 (19–78)	0.336*^a^*	55.0 (19–80)	57.0 (19–74)	0.57*^a^*
**Sex**				0.71*^b^*			0.85*^b^*
Male	40 (66.7)	16 (40.0)	24 (60.0)		25 (62.5)	15 (37.5)	
Female	20 (33.3)	7 (35.0)	13 (65.0)		12 (60.0)	8 (40.0)	
**Stage**				0.15*^c^*			<0.001*^c^*
I	29 (48.3)	14 (48.3)	15 (51.7)		27 (93.1)	2 (6.9)	
II	6 (10.0)	3 (50.0)	3 (50.0)		4 (66.7)	2 (33.3)	
IV	25 (41.7)	6 (24.0)	19 (76.0)		6 (24.0)	19 (76.0)	
**LN involvement**				0.81*^c^*			0.04*^c^*
None	39 (65.0)	16 (41.0)	23 (59.0)		28 (71.8)	11 (28.2)	
Regional	11 (18.3)	4 (36.4)	7 (63.6)		6 (54.5)	5 (45.5)	
Distant	10 (16.7)	3 (30.0)	7 (70.0)		3 (30.0)	7 (70.0)	
**Type of disease**				0.14*^c^*			0.023*^b^*
Nasal	46 (76.7)	20 (43.5)	26 (56.5)		32 (59.6)	14 (30.4)	
Non-nasal	14 (23.3)	3 (21.4)	11 (78.6)		5 (35.7)	9 (64.3)	
**Extranodal involvement**				0.001*^c^*			<0.001*^c^*
1	39 (65.0)	20 (51.3)	19 (48.7)		33 (84.6)	6 (15.4)	
≥2	21 (35.0)	3 (14.3)	18 (85.7)		4 (19.0)	17 (81.0)	
**Presence of B symptoms**	13 (21.7)	1 (7.7)	12 (92.3)	0.01*^c^*	3 (23.1)	10 (76.9)	0.001*^c^*
**Lactate dehydrogenase**				0.16*^b^*			<0.001*^b^*
Normal	35 (58.3)	16 (45.7)	19 (54.3)		29 (82.9)	6 (17.1)	
Increased	25 (41.7)	7 (28.0)	18 (72.0)		7 (28.0)	18 (72.0)	

WB, whole blood; LN, lymph node.

a Wilcoxon rank-sum test.

b Chi-squared test.

c Fisher’s exact test.

### Clinicopathologic Features According to Pre-Treatment WB and Plasma EBV-DNA Status

**Figure 1:**
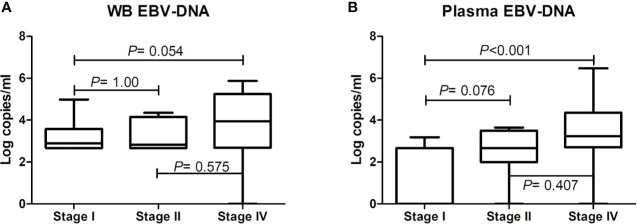
Comparative analysis of pre-treatment Epstein-Barr virus (EBV)-DNA loads in whole blood and plasma according to disease stage. EBV-DNA levels in **(A)** whole blood and **(B)** plasma according to disease stage. The bars show mean values and the error bars show standard errors.

EBV-DNA was detected in pre-treatment WB and plasma in 37 (61.7%) and 23 (38.3%) patients, respectively (**Table 1**). Discordant results were noted in 16 (26.7%) patients, 15 of whom had positive WB but negative plasma EBV-DNA (**Table 2**). Positivity of WB EBV-DNA was significantly associated with the involvement of multiple extranodal sites and presence of B symptoms. Positivity of plasma EBV-DNA was significantly associated with advanced-stage, distant lymph node involvement, non-nasal type, involvement of multiple extranodal sites, presence of B symptoms, and increased LDH level. Both WB EBV-DNA titer (R=0.418, *P*<0.001) and plasma EBV-DNA titer (R=0.502, *P*<0.001) showed moderate correlation with LDH level which was statistically significant. Comparative analysis showed that advanced stage was significantly associated with high plasma EBV titer (stage IV vs. stage I, *P*<0.001) but not with WB EBV titer (stage IV vs. stage I, *P*=0.054) (**Figure 1**). The correlation of EBV-DNA titer between WB and plasma was evaluated separately in limited disease and advanced disease. Whereas patients with limited disease showed a weak correlation (R=0.347, *P*=0.041) between WB and plasma EBV titers, those with advanced-stage diseases showed a strong linear correlation (R=0.851, *P*<0.001) (**Figure S1**).

**Table 2:** Epstein-Barr virus (EBV)-DNA status in the whole blood and plasma at diagnosis.

Patient no. (%)		Whole blood EBV-DNA	
		Negative	Positive
Plasma EBV-DNA	Negative	22 (36.7)	15 (25.0)
	Positive	1 (1.7)	22 (36.7)

### Survival Analysis According to Pre-Treatment WB and Plasma EBV-DNA Status

**Figure 2:**
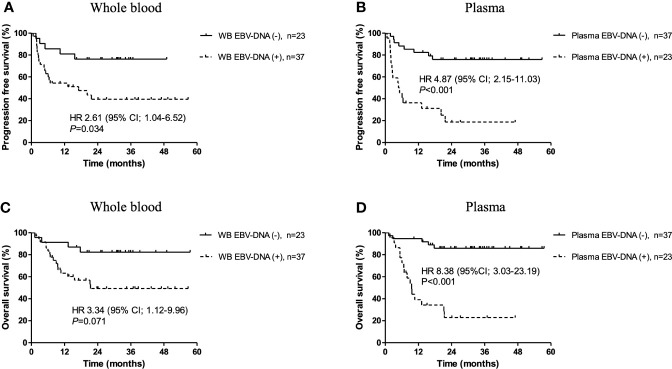
Kaplan-Meier survival estimates stratified by pre-treatment Epstein-Barr virus (EBV)-DNA status. Progression-free survival according to **(A)** whole blood and **(B)** plasma EBV-DNA status at diagnosis. Overall survival according to **(C)** whole blood and **(D)** plasma EBV-DNA status at pre-treatment.

**Figure 3:**
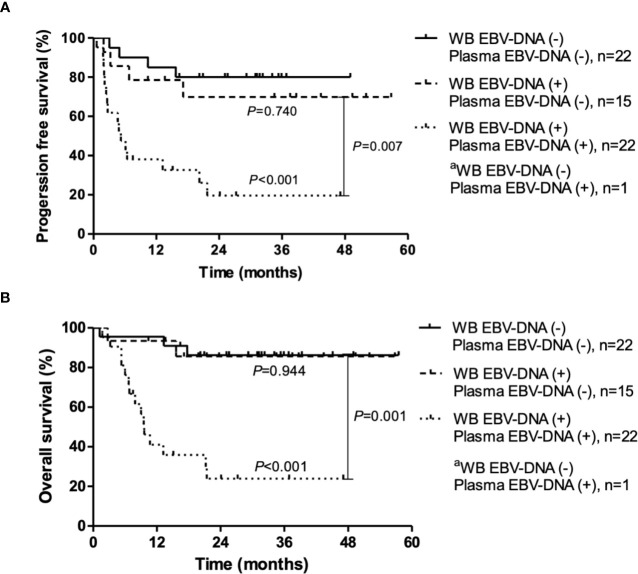
Progression-free survival and overall survival according to pre-treatment Epstein-Barr virus (EBV) status in whole blood (WB) and plasma. **(A)** Progression-free survival. **(B)** Overall survival. P-values were calculated for log-rank comparison of the curves. ^a^Because only one patient was WB EBV-DNA (−) and plasma EBV-DNA (+), the survival curve of this patient is not shown.

During a median follow-up duration of 34.1 months (range, 1.2–57.5), the 2-year PFS and OS rates were 55.0% and 63.0%, respectively. Poor PFS was associated with positivity of pre-treatment WB EBV-DNA (HR, 2.61; 95% CI, 1.04–6.52; *P*=0.034) and plasma EBV-DNA (HR, 4.87; 95% CI, 2.15–11.03; *P*<0.001) (**Figures 2A, B**). Positivity of plasma EBV-DNA at diagnosis was a highly significant prognosticator of OS (HR, 8.38; 95% CI, 3.03-23.19; *P*<0.001). WB EBV-DNA status also showed association with OS, albeit without statistical significance (HR, 3.34; 95% CI, 1.12–9.96; *P*=0.071) (**Figures 2C, D**). Furthermore, the positivity of WB EBV-DNA in those with negative plasma EBV-DNA did not have prognostic values for both PFS (*P*=0.740) and OS (*P*=0.944) (**Figure 3**). In contrast, the positivity of plasma-EBV in those with positive WB-EBV was a significant predictor of both PFS (*P*<0.001) and OS (*P*<0.001). Multivariate analysis adjusting for variables included in the PINK index (age > 60, advanced stage, presence of distant lymph node involvement, and non-nasal type), showed that positive plasma EBV-DNA was independently associated with poor PFS (HR, 4.22; 95% CI, 1.79–9.97; P=0.001) and OS (HR, 8.39; 95% CI, 3.03–23.19; P=0.002), whereas no significant association was observed between positive WB EBV-DNA and PFS (HR, 1.64; 95% CI, 0.71–3.78; P=0.243) or OS (HR, 0.57; 95% CI, 0.10–3.23; P=0.524) (**Table 3**).

**Table 3:** Univariable and multivariable analyses for progression-free survival and overall survival.

Pre-treatmentEBV-DNA Status	PFS				OS				
	Crude		Adjusted^a^		Crude		Adjusted^a^		
	Hazard ratio (CI)	*P-*value	Hazard ratio (CI)	*P-*value	Hazard ratio (CI)	*P-*value	Hazard ratio (CI)	*P-*value	
**WB (+)**	2.61 (1.04–6.52)	0.041	1.64 (0.71–3.78)	0.243	3.34 (1.12–9.97)	0.071	0.57 (0.10–3.23)		0.524
**Plasma (+)**	4.87 (2.15–11.03)	<0.001	4.22 (1.79–9.97)	0.001	8.39 (3.03–23.19)	<0.001	8.39 (3.03–23.19)		0.002

a Adjusted for PINK index; age > 60, advanced stage, distant lymph node involvement, and non-nasal type.

WB, whole blood; PFS, progression-free survival; OS, overall survival; CI, confidence interval; PINK, prognostic index of natural killer lymphoma.

**Figure 4:**
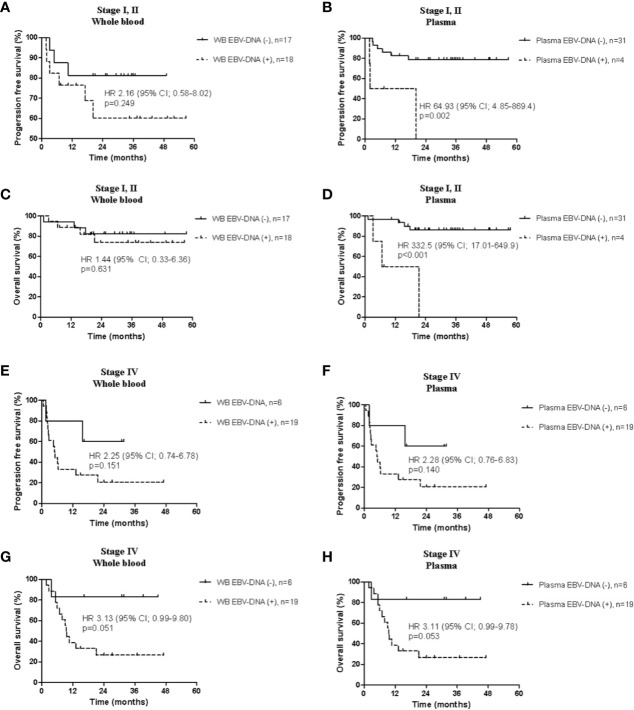
Comparison of survival analysis between the whole blood and plasma in limited stage and advanced stage. Progression-free survival according to **(A)** whole blood and **(B)** plasma Epstein-Barr virus (EBV)-DNA, and overall survival according to **(C)** whole blood and **(D)** plasma EBV-DNA in limited disease. Progression-free survival according to **(E)** whole blood and **(F)** plasma EBV-DNA, and overall survival according to **(G)** whole blood and **(H)** plasma EBV-DNA in advanced disease.

In patients with limited disease, the positivity of plasma EBV-DNA was significantly associated with both poor PFS (HR, 64.93; 95% CI, 4.85–869.40; *P*=0.002) and OS (HR, 332.5; 95% CI, 17.01–649.9; *P*≤0.001), whereas WB EBV-DNA did not show significant associations (PFS: HR, 2.16; 95% CI, 0.58–8.02; *P*=0.249; OS: HR, 1.44; 95% CI, 0.33–6.36; *P*=0.631) (**Figures 4A–D**). In patients with advanced disease, positivity of WB and plasma EBV-DNA showed trends toward poor PFS (WB: HR, 2.25; 95% CI, 0.74–6.78; *P*=0.151; plasma: HR, 2.28; 95% CI, 0.76–6.83; *P*=0.140) and OS (WB: HR, 3.13; 95% CI, 0.99–9.80; *P*=0.051; plasma: HR, 3.11; 95% CI, 0.99–9.78; *P*=0.053), albeit without statistical significance (**Figures 4E–H**).

As an exploratory analysis, we stratified the cohort according to the PINK-E index with WB and plasma EBV-DNA (**Figure S2**). Both WB and plasma EBV-DNA were effective in discriminating between the low- and high-risk groups in terms of PFS (WB: HR, 4.08; 95% CI, 1.65–10.07; *P*=0.002; plasma: HR, 4.45; 95% CI, 1.86–10.65; *P*=0.001) and OS (WB: HR, 7.29; 95% CI, 2.30–23.10; *P*=0.001; plasma: HR, 7.14; 95% CI, 2.46–20.75; *P*<0.001). Moreover, plasma EBV-DNA effectively discriminated between low- and intermediate-risk groups in terms of PFS (HR, 2.43; 95% CI, 1.01–7.25; *P*=0.043) and OS (HR, 5.03; 95% CI, 1.45–17.45; *P*=0.011). However, WB EBV-DNA did not significantly discriminate between the low- and intermediate-risk groups in both PFS (HR, 1.54; 95%CI, 0.53–4.43; *P*=0.428) and OS (HR, 3.18; 95% CI, 0.90–11.27; *P*=0.074).

### Survival Analysis According to Post-Treatment WB and Plasma EBV-DNA

Twenty-seven patients were evaluable for EBV-DNA load after treatment completion; EBV-DNA was detected in the WB of 15 (55.5%) patients and the plasma of 6 (22.2%) patients (**Table S2**). Positivity of post-treatment WB EBV-DNA (HR, 3.95; 95% CI, 1.08–14.46; *P*=0.038) and plasma EBV-DNA (HR, 6.19; 95% CI, 1.94–19.76; *P*=0.002) were both significantly associated with poor PFS (**Figures S3A, B**). While positivity of plasma-EBV at post-treatment was significantly associated with poor OS (HR 8.31, *P*=0.001), there was no significant association between WB-EBV and OS (HR, 3.87; 95% CI, 2.28–30.27; *P*=0.087) (**Figures S3C, D**). Among those with negative post-treatment plasma-EBV (n=21), WB-EBV positivity (n=9) was not associated with both PFS (HR, 2.51; 95% CI, 0.60-10.55; *P*=0.169) and OS (HR, 1.87; 95% CI, 0.31–11.18; *P*=0.488) (**Figure S4**). In contrast, in patients with positive post-treatment WB-EBV, plasma-EBV positivity was significantly associated with both poor PFS (HR, 3.69; 95% CI, 1.02-13.27; *P*=0.035) and OS (HR, 6.07; 95% CI, 1.37–26.86; *P*=0.008).

### Dynamic Changes in EBV Loads in WB and Plasma After Treatment Completion

**Figure 5:**
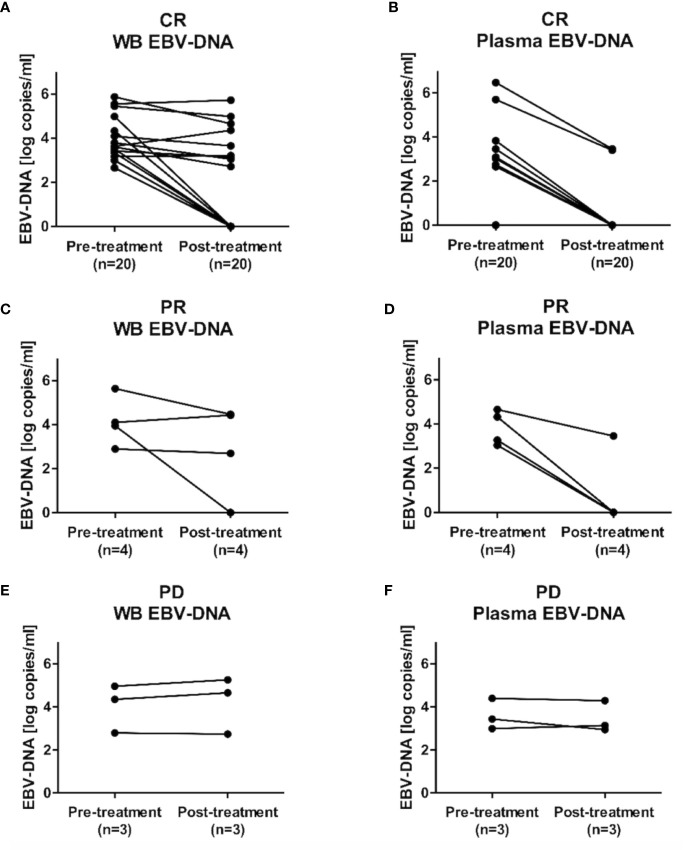
Dynamic changes in Epstein-Barr virus (EBV)-DNA load after treatment completion. Changes in EBV-DNA levels in **(A)** whole blood and **(B)** plasma EBV-DNA in patients with complete response (CR), **(C)** whole blood and **(D)** plasma EBV-DNA in patients with partial response (PR), and **(E)** whole blood and **(F)** plasma EBV-DNA in patients with progression disease (PD).

Dynamic changes in EBV loads after treatment were evaluated in 27 patients (CR: n=20; PR: n=4; PD: n=3) who were evaluable for post-treatment EBV-DNA status. Among patients who achieved CR (n=20), post-treatment WB or plasma EBV-DNA remained negative for all patients who were negative for pre-treatment WB (n=5) or plasma EBV-DNA (n=13), respectively. The negative conversion rates of WB and plasma EBV-DNA were 40.0% (6 out of 15 patients) and 71.4% (five out of seven patients), respectively (**Figure 5**). Among patients who achieved PR (n=4), the negative conversion rates of WB and plasma EBV-DNA were 25% (one out of four patients) and 75% (three out of four patients), respectively. Negative conversions of WB or plasma EBV-DNA were not observed in patients with PD (n=3).

## Discussion

Controversies exist as to which blood compartment is optimal for the measurement of EBV-DNA in ENKTL. The current study is the largest study to date that directly compares the prognostic value of plasma EBV-DNA versus WB EBV-DNA in patients with ENKTL of all stages. Between the two types of blood compartments, only the presence of EBV-DNA in plasma was independently associated with poor survival outcomes in terms of both PFS and OS.

EBV-DNA can be quantified from three compartments in the peripheral blood—plasma, peripheral blood mononuclear cells (PBMC), and WB. Several studies have reported that the plasma has a lower sensitivity than WB in detecting EBV-DNA, which is an expected result because WB contains EBV-DNA present in both PBMCs and plasma (6, 9). In line with the results of previous studies, the sensitivity for EBV-DNA detection was higher in pre-treatment WB samples than in plasma samples in the current study. Whereas patients with advanced diseases showed a high concordance rate between the positivity rates of pre-treatment WB and plasma EBV-DNA, patients with limited diseases showed a high discrepancy. Intriguingly, due to this difference in the sensitivity for EBV-DNA detection, pre-treatment plasma EBV-DNA showed a good correlation with disease stage while WB EBV-DNA did not. This good correlation between pre-treatment plasma EBV-DNA and disease stage explains the relatively low positivity rate of pre-treatment plasma EBV-DNA in the current study compared to that reported in previous study by Ito et al (pre-treatment plasma EBV-DNA positivity rate of 38.3% vs. 57.7%) (9). In the current study, 58.3% of patients had limited disease, whereas only patients with advanced disease were included in the previous study (9). Taken together, our results suggest that plasma EBV-DNA may be a more appropriate surrogate biomarker for tumor burden in patients with ENKTL than WB EBV-DNA.

In our univariate and multivariate analyses, positivity of pre-treatment plasma EBV-DNA was significantly associated with poor PFS and OS. In contrast, pre-treatment WB EBV-DNA was not an independent prognostic factor for PFS and OS. In addition, there was no difference in survival outcomes according to the positivity of WB-EBV DNA in patients who were negative for plasma EBV-DNA. This is in contrast with the results of a previous study, which reported that WB EBV-DNA is superior to plasma EBV-DNA in predicting the prognosis of patients with ENKTL (9). However, this study only evaluated EBV DNA in patients with advanced ENKTL, whereas our current study encompassed all disease stages in evaluating the prognostic value of EBV-DNA in the peripheral blood. In addition, only plasma EBV-DNA was significantly associated with survival outcomes in those with limited diseases. These results suggest that plasma might be the optimal sample for examining the pre-treatment EBV-DNA in patients with ENKTL, considering the inferior prognostic value of WB EBV-DNA in those with limited diseases.

Similar results were observed with post-treatment EBV-DNA, with post-treatment plasma EBV-DNA being significantly associated with poor PFS and OS. This is in line with a previous study, which reported that post-treatment plasma EBV-DNA is a reliable prognostic factor in early-stage ENKTL (20). Our results show that post-treatment plasma EBV-DNA has prognostic values not only in patients with limited disease but also in patients of all stages. In addition, post-treatment plasma EBV-DNA was highly correlated with treatment response, and negative conversion of plasma EBV-DNA was observed in most of the patients with treatment responses. In contrast, post-treatment WB EBV-DNA showed less significant correlation with treatment responses.

The superior prognostic value of plasma EBV-DNA over WB EBV-DNA observed in the current study may be largely attributable to the presence of PBMC EBV-DNA in the WB. Following primary EBV infection, the virus establishes a latent reservoir in resting memory B lymphocytes, which constitute a large proportion of EBV-DNA measured in the PBMCs (21). The vast majority of the global population are infected with EBV, and EBV-DNA can be detected in the PBMCs of almost all of them (15). In contrast, circulating EBV-DNA in the plasma is mainly derived from necrotic or apoptotic tumor cells in those with EBV-positive tumors (12–14). Thus, plasma EBV-DNA would be a more specific marker for tumors compared with PBMC EBV-DNA. Indeed, a previous study has reported that plasma EBV-DNA is a better indicator of tumor burden and prognosis than PBMC EBV-DNA in patients with ENKTL (6).

This study has several limitations. Data regarding post-treatment EBV-DNA was only available in approximately half of the patients in the database. In addition, because EBV-DNA was not serially followed up after treatment completion, the association between disease recurrence and WB or plasma EBV-DNA could not be evaluated. Despite these limitations, this study has several strengths. The results of this study were based on one of the largest patient populations to date, in which the prognostic values of plasma and WB EBV-DNA were directly compared within the same patients. In addition, patients with all stages of ENKTL were included in this study, thus providing a comprehensive evaluation on the impact of plasma and WB EBV-DNA on predicting survival outcomes. Moreover, the results of this study can be directly applied to PINK-E and guide treatment decisions in daily clinical practice.

In conclusion, we showed that plasma is more appropriate than WB in evaluating EBV-DNA in patients with ENKTL. Pre-treatment plasma EBV-DNA had a high prognostic value and yielded information beyond the PINK or its components. Post-treatment plasma EBV-DNA was also associated with poor survival outcomes and may serve as an indicator of the need for additional therapy.

## Data Availability Statement

'The raw data supporting the conclusions of this article will be made available by the authors, without undue reservation.

## Ethics Statement

The studies involving human participants were reviewed and approved by Asan Medical Center, Seoul, South Korea. Written informed consent for participation was not required for this study in accordance with the national legislation and the institutional requirements.

## Author Contributions 

DY designed the work. AJ, YL, S-wL, J-SR, EC, KK, JH, and C-SP collected the data. JH, HC, HS, D-JK, S-OK, and DY analyzed the data. JH and HC wrote the article. All authors contributed to the article and approved the submitted version.

## Funding 

This study was supported by grants (2019-705 to DY) from the Asan Institute for Life Sciences, Asan Medical Center, Seoul, South Korea.

## Conflict of Interest

The authors declare that the research was conducted in the absence of any commercial or financial relationships that could be construed as a potential conflict of interest.
