# Supplementary material for: Superiority of Epstein-Barr Virus DNA in the Plasma Over Whole Blood for Prognostication of Extranodal NK/T Cell Lymphoma
Source: Front Oncol. 2020 Nov 30;10:594692. doi: 10.3389/fonc.2020.594692 (PMC7734249; doi:10.3389/fonc.2020.594692)

Supplementary Figures

Figure S1. EBV-DNA titer distribution between whole blood and plasma in (A) limited disease and (B) advanced disease.

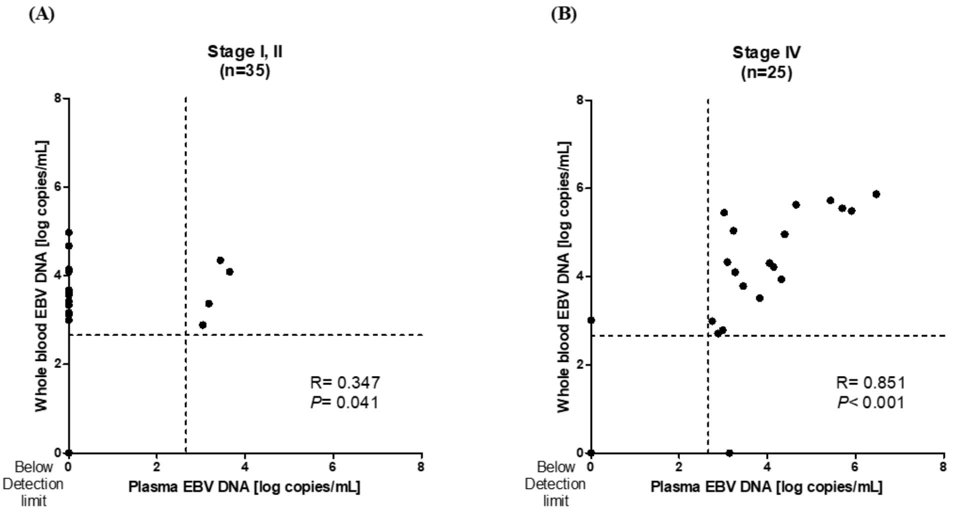

Figure S2. Progression-free survival by PINK-E risk group stratified with (A) whole blood and (B) plasma EBV-DNA. Overall survival by PINK-E risk group stratified with (A) whole blood and (B) plasma EBV-DNA. PINK-E risk factors are composed of age > 60, advanced stage, distant lymph node involvement, and non-nasal type.

(A)

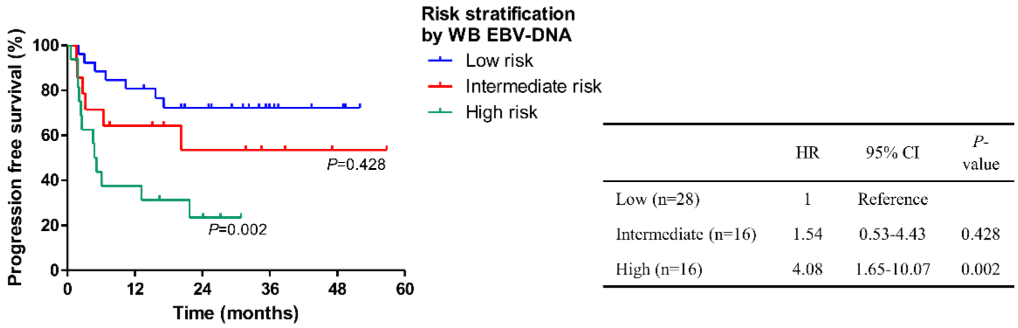

	HR	95% CI	*P*-value
Low (n=28)	1	Reference	
Intermediate (n=16)	1.54	0.53-4.43	0.428
High (n=16)	4.08	1.65-10.07	0.002

(B)

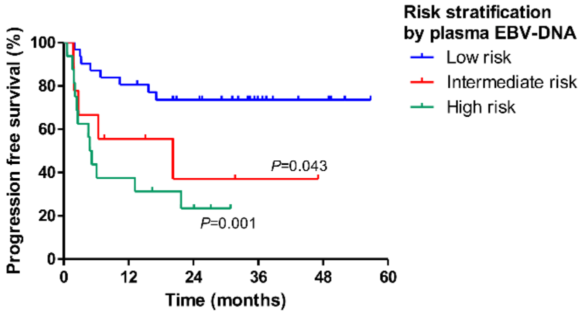

	HR	95% CI	*P*-value
Low (n=34)	1	Reference	
Intermediate (n=10)	2.43	1.01-7.25	0.043
High (n=16)	4.45	1.86-10.65	0.001

(C)

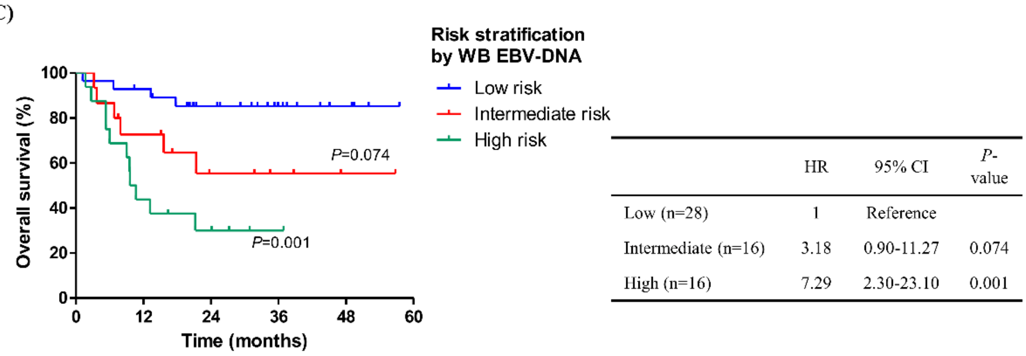

	HR	95% CI	*P*-value
Low (n=28)	1	Reference	
Intermediate (n=16)	3.18	0.90-11.27	0.074
High (n=16)	7.29	2.30-23.10	0.001

(D)

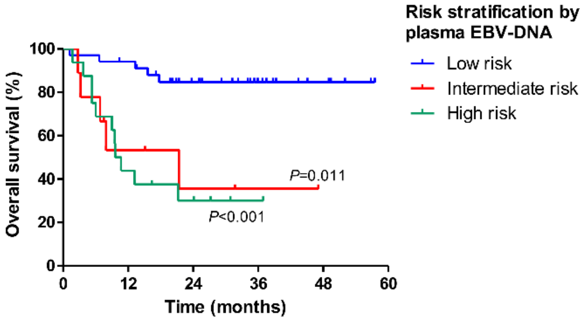

	HR	95% CI	*P*-value
Low (n=34)	1	Reference	
Intermediate (n=10)	5.03	1.45-17.45	0.011
High (n=16)	7.14	2.49-20.75	<0.01

Figure S3. Kaplan-Meier survival estimates stratified by post-treatment EBV-DNA status. Progression-free survival according to (A) whole blood and (B) plasma EBV-DNA status at post-treatment. Overall survival according to (C) whole blood and (D) plasma EBV-DNA status at post-treatment.

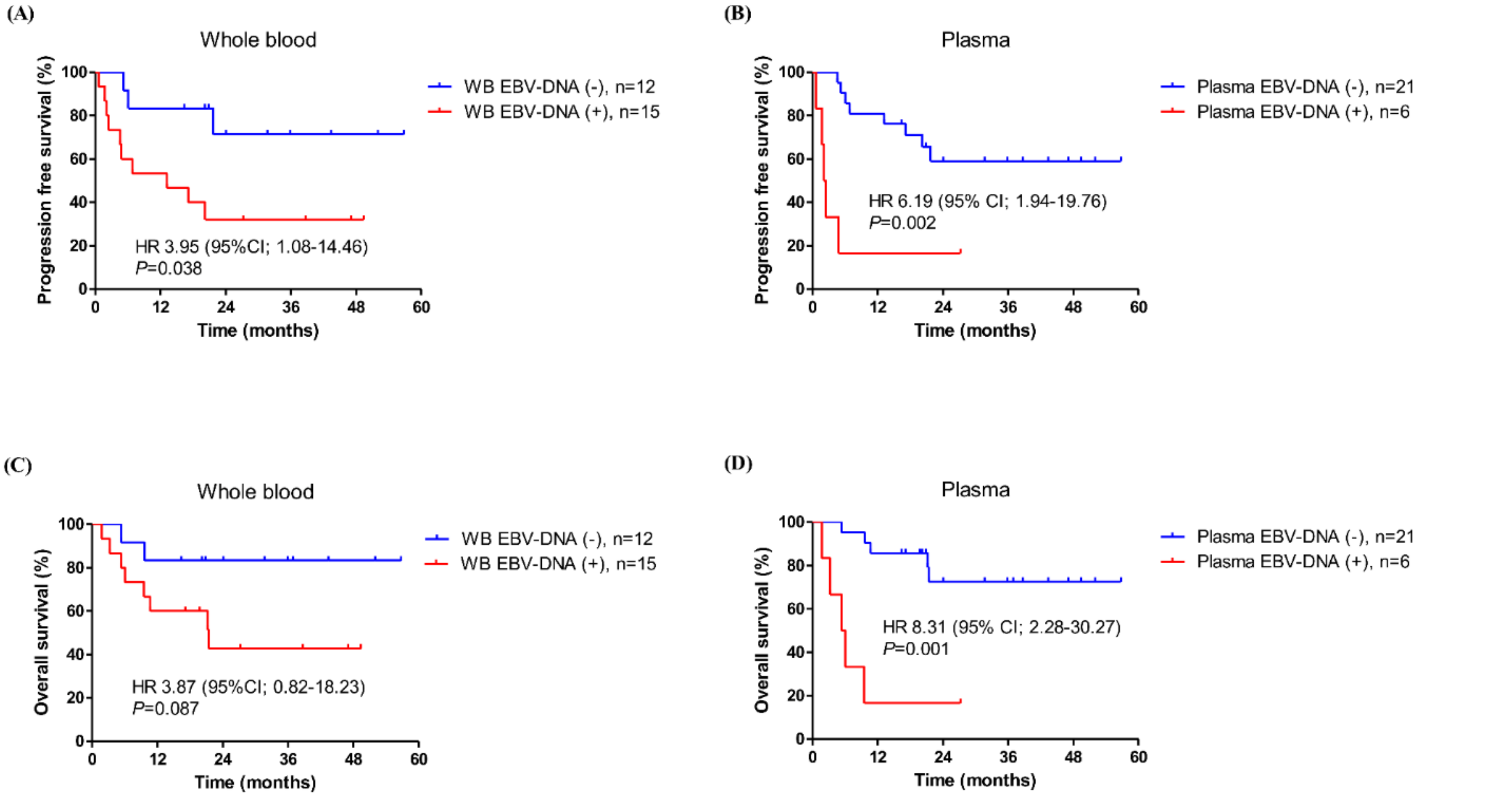

Figure S4. Progression-free survival and overall survival according to post-treatment EBV status in whole blood and plasma. (A) Progression-free survival. (B) Overall survival.

(A)

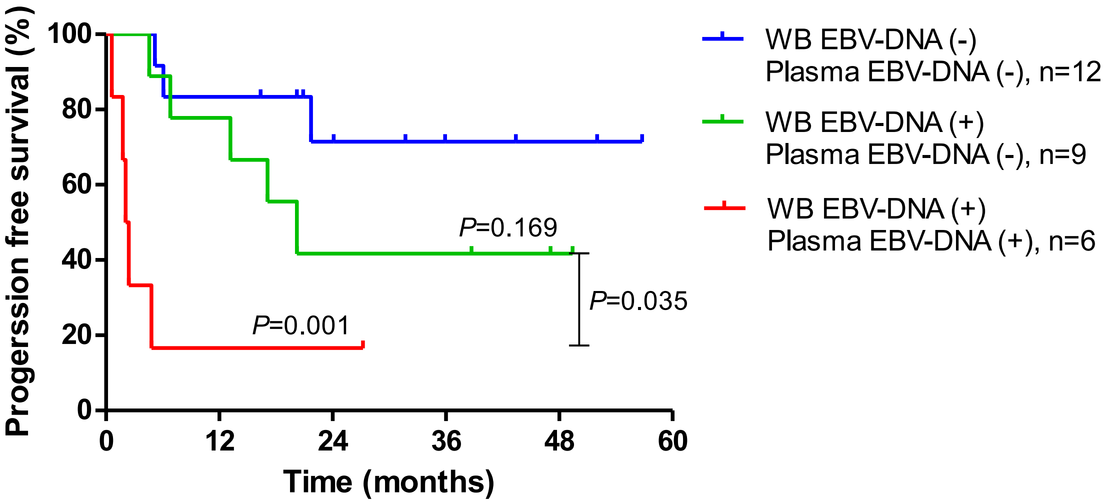

(B)

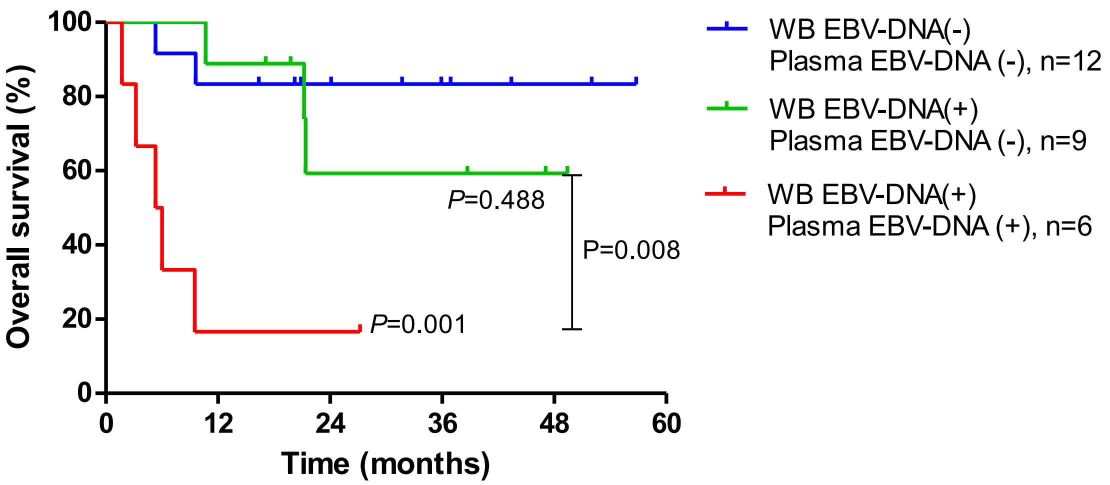

Supplement: Supplementary file 1 [file DataSheet_1.pdf]
